# Traditional medicine applied by the Saraguro *yachakkuna*: a preliminary approach to the use of sacred and psychoactive plant species in the southern region of Ecuador

**DOI:** 10.1186/1746-4269-10-26

**Published:** 2014-02-24

**Authors:** Chabaco Armijos, Iuliana Cota, Silvia González

**Affiliations:** 1 Department of Chemistry, Universidad Técnica Particular de Loja, P. O. Box 11-01-608, Loja, Ecuador

**Keywords:** San Pedro cactus, Medicinal plants, Saraguro, Psychoactive plants, *Yachakkuna*, Healing rituals

## Abstract

**Background:**

During the colonial period, the indigenous saraguros maintained their traditions, knowledge, and practices to restore and preserve the health of their members. Unfortunately, many of their practices and medicinal resources have not been documented. In this study, we sought to document the traditional healers’ (*yachakkuna* saraguros) knowledge about medicinal and psychoactive plants used in the *mesas* and in magical-religious rituals. The study was conducted under a technical and scientific cooperation agreement between the Universidad Técnica Particular de Loja (UTPL), the Dirección Provincial de Salud de Loja (DPSL), and the Saraguro Healers Council (Consejo de Sanadores de Saraguro).

**Methods:**

For the present study, the DPSL and Saraguro Healers Council selected the 10 *yachakkuna* most recognized for their knowledge and their use of sacred and psychoactive species. Ten interviews with the selected *yachakkuna* were conducted between 2010 and 2011 to ascertain how the Saraguro traditional healing system is structured and to obtain a record of the sacred and medicinal plant species used to treat supernatural diseases and for psychoactive purposes.

**Results:**

The present study describes the traditional health system in the Saraguro indigenous community located in southern Ecuador. It also describes the main empirical methods used to diagnose diseases: direct physical examination of the patient, observation of the patient’s urine, documentation of the patient’s pulse, *limpia*, palpation and visionary methods, including supernatural diseases (*susto*, *vaho de agua*, *mal aire*, *mal hecho*, *shuka*) and reports of the use of sacred and medicinal psychoactive plants, such as the San Pedro cactus (*Echinopsis pachanoi*), *wandug* (*Brugmansia* spp.), and tobacco (*Nicotiana* spp.). This study also describes the rituals (*limpia*, *soplada*) employed by the Saraguro *yachakkuna* to treat supernatural diseases. Finally, we report on the main plants used during *limpia* in the Saraguro community.

**Conclusion:**

The current traditional health system in the Saraguro community is the cultural expression of the Saraguros’ presence as an Andean group in southern Ecuador: it represents their character as indigenous group, their ability to survive as a community despite strong external pressure, and the desire to maintain their ancient healing heritage.

## Background

The use of medicinal plants by several indigenous communities in South America, and particularly the use of hallucinogenic plants with therapeutic purposes, is linked to the shamanism that the ancient hunters brought on their journey from Northeast Asia to the modern-day American continent [1,2]. Ancient civilizations that migrated from Asia and settled in America believed that diseases are closely linked to spirits and esoteric rituals. Thus, shamanic practices were closely related to the use of hallucinogenic species that allowed the shamans to reach several states of consciousness or visionary experiences, in a way similar to that of current shamans from different indigenous communities in South America [3,4]. In the Andean region, these healers are known as *yachak* or *yachakkuna*[5].

According to Cabieses [6], three types of consumption have been identified and vary according to the location in the South American Andes, the type of plants consumed, and the mechanism of action exerted by psychoactive chemicals: (i) *mescalinismo* (the consumption of cactus containing mescaline, such as *Echinopsis pachanoi*) in Andean valleys and desert areas of the coast; (ii) *cocainism* (the use of *Erythroxylum coca*) in the valleys and plateaus of the Andes; and (iii) *harminism* (the consumption of plants containing harmine, such as the *ayahuasca* beverage) in the Amazon.

**Figure 1:**
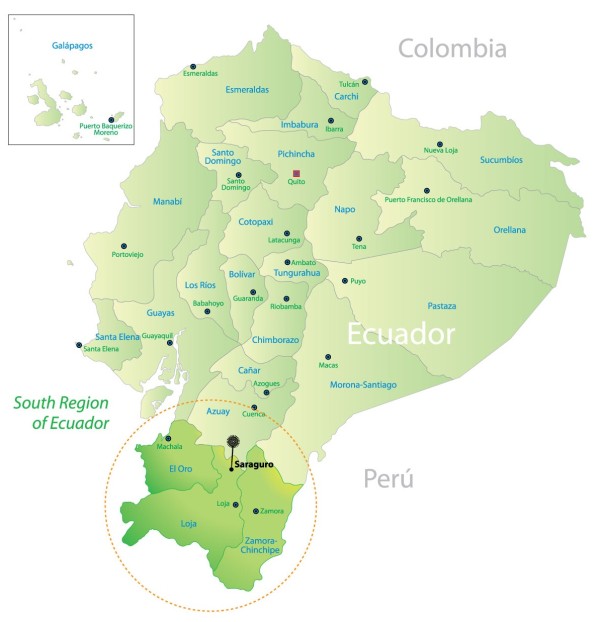
Southern region of Ecuador.

In the Ecuadorian Andes, the use of plants as therapeutic agents is an important feature of traditional medicine and is still practiced in many indigenous communities. A noteworthy example is the Saraguro ethnic group, which belongs to the Kichwa community of Ecuador. The Saraguro group is located in the southern region of the country (Loja and Zamora-Chinchipe provinces), as shown in Figure 1. This region is known as the area with the greatest biodiversity on the planet; it contains approximately 4,000 species of vascular plants [7,8] and is located in the Podocarpus National Park. In the province of Loja, the Saraguro community lives in the canton that bears its name.

The Saraguro community has a population of approximately 60,000 inhabitants. They are considered one of the most organized ethnic groups in Ecuador, and they preserve their ancestral knowledge and technology, culture, medicine, language, and customs [9-11]. Currently, the majority of Saraguro community members speak *runashimi* (the Kichwa language) and Spanish, whereas the Saraguros who inhabit remote areas, such as Tambopamba, Gera, or Oñacapac, speak only Kichwa.

The origin of the Saraguros as an indigenous community was not determined until recently. Some theories claim that the Saraguros are *mitimaes,* part of a conquered population forced by the Incas to settle in a distant area. For this reason, they were mobilized by the Incas as part of a strategy to ensure social peace within the Inca Empire. According to Rowe [12], a small group of *paltas* (native inhabitants of Loja) was transferred to Bolivia and, at the same time, a number of the inhabitants of the Bolivian Altiplano were transferred and established in the current Saraguro’s locations. In Kichwa language, Saraguro means “land of corn”. Among Saraguro communities, family is the basis of their cultural identity: their beliefs, traditions, and customs as well as the use of medicinal plants and traditional knowledge are transmitted orally among family members [13].

Over the years, the Saraguro population has used a broad variety of medicinal plants [10,14]. Previous studies of the Saraguro community describe the existence of *hampiyachakkuna* (wise men or women) who have knowledge of plants’ healing properties [9,15,16]. These people use their wisdom to heal diseases in accordance with the medical traditions of other Andean communities. *Yachakkuna* are responsible for diagnosing, treating, and healing physical diseases and other disorders that have a supernatural character.

Although previous studies have considered the use of hallucinogenic and sacred species plants by Ecuadorian *yachakkuna*[17,18], there are no reported studies on traditional Saraguro medicine. Considering the lack of ethnobotanical information on the Saraguros medical traditions, the current research describes the empirical healing methods that are used by this community. The aim of the present study is to provide data on plant species and other natural resources that are used during healing rituals and have magical and religious significance. This research is part of a strategy that seeks to rescue and validate ethnic consciousness and to promote the sustainable use of Saraguro medicinal and biological resources.

## Methods

The study was conducted under a technical and scientific cooperation agreement between the Universidad Técnica Particular de Loja (UTPL), the Dirección Provincial de Salud de Loja (DPSL), and the Saraguro Healers Council (Consejo de Sanadores de Saraguro) to recover and recognize the traditional knowledge of herbal medicinal resources used by the Saraguro community. For the present study, the DPSL and Saraguro Healers Council selected the 10 *yachakkuna* most recognized for their knowledge and use of sacred and psychoactive species. In recent years, 65 indigenous Saraguro *yachakkuna* have been registered by the health department because they have been working for several years in the region of Saraguro; such support has been crucial for the development of this study. The interviewed healers were two women and eight men aged 65 to 80 years old.

The selected *yachakkuna* worked daily in agriculture, in animal care, and at home. Three of them had no education of any kind, two of them attended primary school, four were enrolled in secondary school but did not complete it, and one attended university and worked as a teacher in a bilingual school in the Saraguro city.

These *yachakkuna* have undergone a training process for most of their lives. Nine of them obtained knowledge of traditional medicine from their grandfather, father, mother, or another close relative. One of them was trained by an old *yachak* from his community without any interference from his family. They all have participated in at least one healing experience conducted by a *yachak* from another Andean indigenous community in Ecuador, such as Cañar, Chimborazo, Imbabura, or Pichincha. Two of the *yachakkuna* gained experience by working with Shuar shamans from the Amazonian region of Ecuador and shamans from southern Ecuador and northern Perú. Another fact that validates their experience is their participation as leaders in the *raymis* rituals, celebrations, and other ceremonies in Saraguro and other communities. One of the *yachak* has been accredited by the DPSL and sent to attend ceremonies and exhibitions as a representative outside of Ecuador.

Ten interviews with the selected *yachakkuna* were conducted between 2010 and 2011 to describe how the Saraguro traditional healing system is structured and to obtain a record of the sacred and medicinal plant species used to treat supernatural diseases and for psychoactive purposes. Information about extract preparation, the plant part used, and the manner of application or administration of plant species was obtained from these surveys. With the permission of each *yachakkuna,* photographs of each plant species were taken to create a digital database of these plants. A photographic record of the ceremonies and healing rituals was also created.

The obtained data were gathered in tables containing ethnobotanical information regarding the plants’ common name in Kichwa, scientific name, medicinal use, preparation, and administration. The interviewed *yachakkuna* were brought together to establish an accurate definition of the concepts of health and disease; during these sessions, it was decided that the Kichwa name should be used for some supernatural diseases.

The plants were collected in three different locations where the *yachakkuna* typically collect them (Table 1). The collected samples were identified in the Universidad Nacional de Loja (UNL) Herbarium with the collaboration of the Bolivar Merino curator of the herbarium. Reference samples were deposited in the Universidad Técnica Particular de Loja (UTPL) Herbarium with their own identification number. The systematics and nomenclature of the species reported in the study were based on the Catalogue of the Vascular Plants of Ecuador (Jørgensen & Leon-Yanez, 1999).

**Table 1:** Coordinates of the plant collecting locations in the Saraguro regions

**Location**	**Coordinates**		
*Achupallas*	17694877E	9594739 N	2,890 mamsl
*Sunin*	17694877E	9594739 N	3,128 mamsl
*Fierro Urku*	17686161E	9589043 N	3,621 mamsl

This work was realized with the support of the Secretaria Nacional de Ciencia y Tecnología del Ecuador (SENESCYT) and the permission of the Ministry of the Environment of Ecuador.

## Results and discussion

### Traditional healthcare system in Saraguro

The healthcare system in Saraguro is based on the knowledge of the *yachakkuna,* who can be classified into four categories depending on their expertise: (i) the *wachakhampiyachak* (midwife), who uses plants and natural remedies to cure diseases during prenatal care, birth, postpartum and in the early years of a baby’s life; (ii) the *yurakhampiyachak*, who uses garden or wild plants to cure diseases that have organic symptoms, such as headache or fever; (iii) the *kakuyhampiyachak* (a person who treats bone and joint disorders), who prepares bandages and lotions with plant extracts and animal fats to cure muscle problems (sprains) and broken bones; (iv) the *rikuyhampiyachak* (authentic shaman), who uses hallucinogenic and psychoactive plants to cure supernatural diseases during sessions that are known as *mesas* or *mesadas* (rituals with religious and magical significance) [15,19,20].

In the Saraguro community, the *hampiyachakkuna* are accredited as such by their own community based on their experience and success in treating and curing diseases. During the 1970s, small health centers and subcenters of the government healthcare system were created in Saraguro; therefore, today there are two types of healthcare systems: the traditional system typical of Saraguro community, which is composed of *yachak*; and the conventional health system, which consists of physicians (specialist or general physicians) and nurses.

Currently, the traditional healing methods used in Saraguro community consist of a mixture of indigenous, Spanish, and modern practices. Thus, if the *yachak* establishes that the disease cannot be treated using the empirical method, they suggest using modern medical methods.

A previous study showed that in 2009, only 30 cases out of 47 were treated at health subcenters in Saraguro, meaning that pregnant indigenous women prefer to trust their health and their children’s health to the *wachakhampiyachak*. This is a clear example of the accreditation of the *hampiyachakkuna* by their own community.

At present, the Loja Department of Provincial Health’s Department of Indigenous Health has 65 *yachakkuna* registered for a community of 60,000 members, which can be considered a representative number given the total number of the community members.

According to José Cartuche Quizhpe, a Saraguro community leader, each *yachak* must demonstrate the effectiveness of his cures before being accepted as a healer by the community because the prestige and fame of the *yachakkuna* are based on the effectiveness of their cures. The effectiveness of healing is a filter that prevents a charlatan from claiming to be a *yachak*.

### World view of disease according to Saraguro community

For all the *yachakkuna* consulted, diseases are the result of an imbalance between the individual, his or her immediate environment, and the spiritual world. The *yachakkuna* from this community define the body’s ability to react to diseases as *jinchi* (vital balance); a strong *jinchi* enables the body to resist diseases, whereas a weak *jinchi* makes the body more susceptible to diseases. This view is in agreement with the Andean world view, which states that to enjoy health and wellness, a person must first be in harmony with himself or herself, a state that in Kichwa is known as *allicai*; second, he or she must live in harmony with the others, a condition that is known as *allikawsay*[21].

Generally, disease can be caused by spiritual factors (such as *encanto*, *susto*, *mal aire*, *malos espiritus*) acting autonomously or inflicted by other person. Other factors that cause diseases are transgressions of moral or social norms and organic disorders. Table 2 presents the origins of diseases according to the Andean world view.

**Table 2:** Causes of diseases according to the Andean worldview of health and disease

**Disorder**	**Cause**	**Disease**
Organic disorder	Poor diet, poor intake of food and beverage, alimentary excess	Alteration of the digestive, respiratory, circulatory, nervous or reproductive systems
Psychosocial disorder	Transgression of moral or social norms	Anger, rage, envy, suffering, grief
Supernatural disorder	Negative energy of a person or place	*Shuka,* mal de ojo, *susto*, *encanto*, supernatural diseases

Among the Saraguro members and other different indigenous groups in the Andes, there are diseases that are defined by a specific term [22]; for example, *la peste* (plague) is used to describe various infectious diseases, such as the flu.

### Main diagnostic methods used by the Saraguro community

In Andean medicine, the empirical methods used to describe diseases are simple and straightforward; the necessary data about disease being treated are obtained from the personal dialogue between the patient and *yachak*. Observation, sight, hearing, and touch are important tools used to investigate the patient’s body diagnose the disease.

In indigenous medicine, there are situations in which the diagnosis is followed by treatment in the same manner used in occidental medicine. Nevertheless, in some cases, the diagnosis and treatment take place simultaneously: for example, while cleansing the patient with egg, the *yachak* finds out which part of the body is sick and, at the same time, he or she heals the patient by eliminating negative energy.

Table 3 presents the main ancestral diagnostic practices used in traditional medicine in Saraguro.

**Table 3:** Diagnostic methods used in empirical medicine in the Saraguro community

**Type of diagnosis**	**Description of the method**
Direct physical examination of the patient	The *yachak* observes the patient’s face, eyes, or tongue, for example, when diagnosing *susto* or *espanto*.
Observation of the urine	The *yachak* observes the patient’s urine, for example, when diagnosing an intestinal inflammation.
Patient’s pulse	The *yachak* observes the patient’s pulse; for example, the healer can determine if the patient is worried or has a nervous condition that must be treated.
*Limpia* (cleaning) with an egg	The *yachak* passes a chicken egg over the area to be treated. The egg is then deposited in a glass with water. The healer observes the egg and then he/she establishes the diagnosis, for example, when diagnosing *vaho de aire.*
Palpation	This method is used to determine whether there is any broken bone or muscle contracture. It is also used by the *wachakhampiyachak* (midwife) to determine the position of the fetus during pregnancy.
Visionary methods	This method is used by visionary *yachak.* Under the effect of hallucinogenic species and/or psychoactive preparations, the *yachak* reaches a state of ecstasy. In this state, the healer can determine the problem that affects a person and how to treat it, for example, when diagnosing *shuka* or envy.

### Classification of supernatural diseases

According to the Saraguro world view, supernatural diseases are considered more important than other types of diseases, thus indicating a strong magical and religious concept of health and disease that is still maintained by Saraguro community and has been described in previous studies [19,20,23,24]. Table 4 presents the definition of each supernatural disease according to the Saraguro worldview.

**Table 4:** Definitions of supernatural diseases in the Saraguro community

**Disease**	**Definition**	**Symptomatology**
*Susto*	A disease that is produced by unpleasant experiences, accidents, violent episodes, or moments of distress that produce an emotional impact on the patient.	Nervousness, lack of appetite, sleep loss.
*Vaho de agua*	This disease occurs when the person is exposed to water mist, for example, when crossing a bridge. People who have been beaten or injured or women who have recently given birth are more susceptible to this disease.	Severe pain in the extremities, wounds, or strokes that arise from time to time.
*Mal aire*	A disease caused by strong winds experienced while the person walks down a hill, by contact with cold air when the person leaves a sheltered place, or when a person walks through cemeteries or places where there are hidden treasures.	Dizziness, headache, vomiting, stomach pain. The patient suffers body deterioration.
*Mal hecho*	This disease is caused by damage that a person intentionally inflicts on another individual. The diseases that are manifested are organic and psychological, emphasizing envy and jealousy.	The patient suffers personal misfortunes, accidents, death of loved ones, and financial losses. This leads to depression.
*Shuka*	This disease is the so-called *mal de ojo*. It is caused by a person directed a forceful gaze toward someone else. In this case, the disease occurs without maliciousness. Otherwise, *shuka* occurs with bad intention.	Fainting, nervousness, pale face, headache, sadness, behavioral, and personality changes. Children are more prone to this disease.

Today, it is not common to hear of certain diseases that are believed to be caused by *malos espiritus* because they are considered fantasy or superstition by scientific medicine and by those outside the Saraguro community. Therefore, the present study underlines the fact that valuable ancient medical knowledge is being lost because of acculturation.

According to *yachak* Polibio Japón Cango, some diseases known by elderly Saraguro healers, such as *huairashcamanta* (a disease caused by *malos espiritus* in people who walk by places where people have died), *huatucayashca* (a disease that occurs mostly in pregnant women who travel or sleep in enchanted hills), or *mancharishca* (*susto*), have lost their relevance. This irrelevancy is the result of the acculturation process that the Saraguro members have undergone in the last 50 years. Consequently, the main goal of the present study is to recover ancient Saraguro knowledge.

### Empirical treatment methods

#### *Limpia* in the Saraguro community

*Limpia* (healing treatment) is performed to remove a person’s negative energy and to remove the effect of the *shuka* (the so-called *mal de ojo*). According to Saraguro healers, certain diseases are caused by the negative energy of another person. *Limpia* is the first empirical treatment provided; after *limpia,* a person can heal fully without further necessary treatment. Table 5 presents some plants that are considered powerful for this treatment.

**Table 5:** **Main plants used during *limpia *** **in the Saraguro community**

**Common name**	**Scientific name**	**Species voucher**	**Part used**
*Santa María silvestre*	*Diplostephium macrocephalum* S.F. Blake	PPN-pi-009	The entire plant
	ASTERACEAE		
*Santa María de huerta*	*Tanacetum parthenium* (L.) Sch. Bip.	PPN-pi-014	The entire plant
	ASTERACEAE		
*Poleo*	*Minthostachys mollis* (Kunth) Griseb.	PPN-pi-007	The entire plant
	LAMIACEAE		
*Poleo de llano*	*Clinopodium sp.*	PPN-pi-004	The entire plant
	LAMIACEAE		
*Shuyu rosas de cerro*	*Eryngium humile* Cav*.*	PPN-pi-013	The entire plant
	APIACEAE		
*Laurel grande*	*Myrica parvifolia* Benth.	PPN-pi-015	Blooming branches
	MYRICACEAE		
*Romero*	*Rosmarinus officinalis* L.	PPN-pi-019	Branches
	LAMIACEAE		
*Ruda*	*Ruta graveolens* L*.*	PPN-pi-010	The entire plant.
	RUTACEAE		
White *wandug*	*Brugmansia candida* Pers.	PPN-pi-021	Branches
	SOLANACEAE		
Pink *wandug*	*Brugmansia suaveolens* (Willd.) Bercht. & J. Presl		Branches
	SOLANACEAE		
Red *wandug*	*Brugmansia sanguinea* (Ruiz & Pav.) D. Don		Branches
	SOLANACEAE		
*Marco cari* and *marco warmi*	*Ambrosia artemisioides* Meyen & Walp	PPN-pi-001	Branches
	ASTERACEAE		
*Cholo valiente*	*Tagetes terniflora* Kunth	PPN-pi-017	Branches
	ASTERACEAE		

During *limpia,* a few branches of each plant are used to form two bunches. One bunch is held in each hand. The cleansing is conducted from the top of the body to the bottom while the healer and patient repeat specific prayers. Depending on the case and the possibility of procuring the indicated species, either all appropriate plants are used or only those that are easily accessible are used.

The plant species used for *limpia* vary depending on the disease; for example, when *limpia* is employed to treat *mal aire,* poleo (*Clinopodium* sp.), turpec (*Solanum oblongifolium* Dunal.), shadán (*Baccharis obtusifolia* Kunth), ajenjo (*Artemisia sodiroi* Hieron.), limoncillo (*Siparuna muricata* (Ruiz & Pav.) A. DC.), laurel (*Myrica parvifolia* Benth.), marco (*Ambrosia artemisioides* Meyen & Walp.), romero (*Rosmarinus officinalis* L.) *ushku sacha* (*Loricaria thuyoides* (Lam.) Sch. Bip.), chilca negra (*Baccharis* sp*.)*, and *cholo valiente* (*Tagetes terniflora* Kunth) are used.

To increase the *limpia*’s effect, an infusion of *chichira* (*Lepidium chichicara* Desv.), ruda (*Ruta graveolens* L.), poleo chico (*Minthostachys mollis* (Kunth) Griseb.), and shadán (*Baccharis obtusifolia* Kunth) is administered.

According to Isabel Medina Tene, Saraguro *yachak*, the cleansing rituals must be performed by people who have good physical, spiritual, and emotional strength, as the power of the healing ritual depends on the nature of the plants used and the person who performs the ritual.

Among the Kichwa peoples of Ecuador (Cañar and Cuenca), *limpia* and a diagnostic method using the Andean guinea pig (*Poronccoy cavia* Pchudii) are very popular. However, according to our research, these methods are not commonly used in the Saraguro region.

#### Soplada

After *limpia,* the *yachak* blows a special preparation known as *cargado* (an alcoholic extract of plants possessing certain power, together with a sugar cane liquor called *aguardiente* and various perfumes, such as Agua Florida) on the person. The composition of *cargados* varies depending on the disease and the *yachak* who prepares them. For example, to treat *shuca,* a *cargado* containing Santa María. (*Tanacetum parthenium* (L.) Sch. Bip., ruda *(Ruta graveolens* L.), duamaric (*Tibouchina laxa* (Desr.) Cogn.), Shuyu rosas (*Eryngium humile* Cav.), Killu rosas (*Tagetes erecta* L.), and cholo valiente (*Tagetes terniflora*) is prepared.

*Cargado* can also be applied by external rubbing on the affected part. For example, in the case of fractures, shock, or muscular strains, a *cargado* containing cararango (*Lobelia* sp.), ruda (*Ruta graveolens* L.), Killu rosas (*Tagetes erecta* L.), tobacco (*Nicotiana* spp.), valeriana (*Valeriana pyramidalis* Kunth), and *cholo valiente* (*Tagetes terniflora* Kunth) is used.

To treat *susto*, after *limpia,* the Saraguro healer blows over the patient a *caragado* called *Ishpingo* that contains cararango (*Lobelia* sp.), Santa Maria (*Tanacetum parthenium* (L.) Sch. Bip.), female and male *Trencilla* (Lycopodiaceae spp.), toronjil (*Melissa officinalis* L.), and Palo santo (*Bursera graveolens* L.). This *cargado* is prepared by maceration in water or *aguardiente*.

In the Saraguro community, it is common for every family to prepare its own *cargado* and use it to treat *mal aire* or *susto*. According to different investigations conducted in South America, this *cargado* is characteristic to the Andean indigenous people [25-27].

### Using plants as a purgative

The use of various purgative plant extracts clears the organism; this purge may be the reason for a person’s healing or may prepare the body for further treatment. *Trencillas* (*Lycopodiaceae* spp.) mixed with other plants, such as cactus *aguacolla* or San Pedrillo *Echinopsis pachanoi* (Britton & Rose) Friedrich & G.D. Rowley are used in purgative preparations.

### Elements used in rituals performed by *yachakkuna*

**Figure 2:**
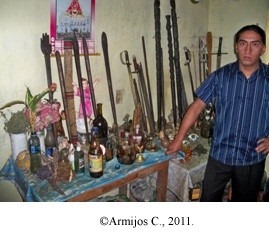
**Orlando Leonidas Gualán, chairman, Council of Healers, Saraguro, beside a healing *mesa *** **in 2011.**

**Figure 3:**
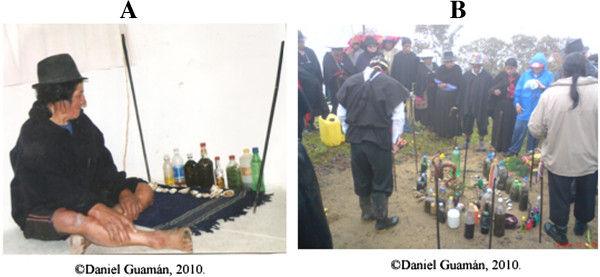
**Saraguro healing *mesas*. (A)** José María Gonzáles, visionary *yachak,* Tambopamba. **(B)** Healing ritual in San Lucas.

In the ritual ceremonies known as *mesas* or *mesadas,* certain elements are used along with sacred plant species [3,17,18,25]; the total number of items reported by Saraguro *yachakkuna* are indicated in Table 6 and Figure 2. The elements used vary according to each *yachak;* some *mesas* are very simple (Figure 3A), and they are held in lakes or enchanted hills (Figure 3B).

**Table 6:** Main items used in healing rituals in the Saraguro community

**Elements of the *mesa***	**Description**
Sword or machete	These items are used as a defense against enemies or negative energies that threaten the *mesa.*
*Bastón de mando*	This item represents the maximum power that is present in the ritual. These canes have magical powers: if they are passed through the body of a person, they clean the person of negative energies.
Stones	Stones serve to cleanse a person and remove his/her negative energies. Some are archaeologically significant and appear to have more power than normal stones.
Shells	Shells are used as vessels for ingesting the psychoactive species extract.
San Pedro cactus beverage	Sacred drink used to achieve an ecstatic state and the desired contact with the gods and the supernatural.
Sugar	Sugar is the symbol of the good, the sweet, the blooming. It is used to prepare a drink with lime juice and carnation flowers that finishes the *mesa* and completes the ritual.
Macerated tobacco	Tobacco leaves are extracted in water or alcohol and perfumes. This extract is inhaled to enhance the effect of the San Pedro beverage.
Extract of wild sacred plants	An extract made from wild sacred plants known as *wamingas* and *trencillas* (*Lycopodiaceae* spp.). It has the power to cure and remove negative energies.
*Aguardiente*	Distilled from sugar cane, this item is used as an offering during the ritual.
Agua Florida and perfumes	These items remove the negative energy during *soplada*. In this manner, the environment, *mesa*, *yachak,* and patients are cleansed.
Holy water	Holy water removes negative energy during the ceremony. For the *yachak,* holy water is pure and fresh water that has been collected from lagoons and sacred lakes.
*Rienda*	This item is a rope made of cattle tail hair. It represents a sacred element that removes negative energy and diseases while the patient passes it through his/her body, simulating an external cleaning.
Candle	In the flame of the candle, some visionary *yachakkuna* can see the health and welfare of a patient. The intensity of the flame indicates the patient’s problem and his/her future.

### Use of psychoactive species

#### Use of San Pedro cactus

San Pedro cactus, also called *aguacolla* or San Pedrillo, *Echinopsis pachanoi* (Britton & Rose) Friedrich & G.D. Rowley, is one of the most common sacred plants employed by Saraguro *yachakkuna.* A*guacolla* is a plant that protects the matrimonial union, the family, and the peaceful coexistence of parents and children; therefore, it is cultivated close to the house.

For hallucinogenic purposes, San Pedro cactus is used as a beverage during magical-religious ceremonies to attain altered states of consciousness that allow the *yachak* to recognize the patient’s condition and determine the possible treatment. According to Madsen [28] and Schultes & Hofmann [3], in South America, the plant has an important cultural history, as the indigenous population of Ecuador and Peru [27,29,30] has used this plant for its hallucinogenic effects since pre-Columbian times.

Another property attributed to the beverage is the purge that clears the organism. Ingesting the beverage may cause vomiting or diarrhea, which removes the agents that affect patient’s health. After drinking the beverage, some people suffer an emotional effect that leads to tears. This emotional release is considered a form of spiritual healing. For several of the consulted *yachakkuna,* whether a person has these symptoms is related to his or her biological and psychological constitution, the provided dose, and the patient’s disease state.

In the Saraguro community, *mesas* with San Pedro cactus are performed for several purposes: (i) to regain health when the person has a certain disease for which conventional medical treatment was not effective; (ii) to gain money; (iii) to regain the love of someone; and (iv) to find lost animals or objects.

Table 7 presents the main applications attributed to this plant in Saraguro community. The reported uses of San Pedro cactus to cure supernatural diseases, such as *shuka* and nervous system alterations, are in agreement with previous studies conducted in Saraguro community [15,16].

**Table 7:** Uses of San Pedro cactus in the Saraguro community

**Uses**	**Preparation**	**Administration**
To induce visions (oral)	The cactus pulp is cooked for 7 h until a viscous consistency is obtained.	It is drunk (one or two glasses).
To induce visions (inhaled)	The cactus is cooked and extracted with *trencillas*, *wuamingas* (*Lycopodiaceae* spp.), tobacco leaves, and Agua Florida.	It is administered through the nose using small shells. The process is led by the *yachak.*
As a purgative	Fresh San Pedro juice is mixed with other plant preparations, known as *cargados.*	The beverage is drunk while the patient is in a fasting state before breakfast; the process is repeated for three days.
To treat *shuka*	The pulp juice is mixed with an extract of tobacco and *cararango* (*Lobelia* sp.).	The extract dose drunk by the patient is approximately 5 ml.
To treat anxiety	One San Pedro leaf is added to 1 liter of infusion prepared with plants that are used to treat anxiety.	The infusion is drunk for several days until the patient recovers.
As an anti-inflammatory or wound disinfectant.	The cooked pulp is used as bandage.	The affected part is washed, and the bandage is placed.

Many Saraguro *yachakkuna* experiment with different types of cactus that, according to their criteria, vary depending on the number of edges; the San Pedro cactuses most commonly used have six, seven, or eight edges and are collected in the surrounding Saraguro areas, such as Loja, Catamayo, and part of the El Oro Province. The most desired type is the seven-edged *Echinopsis pachanoi*, which has a high content of mescaline and other alkaloids. According to Madsen [28], these toxic substances are used by the plant to prevent animals from consuming its branches. The active principle of the San Pedro cactus is mescaline [31]. Although there are no studies on the chemistry of the drink used by Saraguro, the estimated concentration of mescaline in a drink ingested during a traditional medicine session in Perú was 50-70 mg [25].

### Use of floripondios plants

The *Brugmansia* plants are considered sacred by the indigenous peoples of South America [32,33]. The Saraguro *yachakkuna* call these species *wandug* or *floripondios.* According to Schultes & Hofmann [3], the ancient Andean culture knew of at least six *Brugmansia* species. Four distinct species can be found in the Saraguro community area: one with red flowers, called red *wandug* (*Brugmansia sanguinea*); one with yellow flowers (*Brugmansia aurea*); one with white flowers, called white *wandug* (*Brugmansia candida*); and one with pink flowers, called pink *wandug* (*Brugmansia suaveolens*). Some of these species have been previously used in different indigenous populations of Ecuador [34] and Peru [33,35,36]. The use of *B. aurea* and *B. sanguinea* by other Kichwa communities in Central Andean Ecuador as psychoactive and narcotic species has been documented [37,38].

Table 8 presents the uses of these sacred species by the Saraguro *yachakkuna*. Most Saraguro homes have a *floripodio* plant in the garden to protect their inhabitants from negative energies or witchcraft.

**Table 8:** **Uses of *wandug *** **(*Brugmansia *** **spp.) in the Saraguro community**

**Species**	**Common name**	**Traditional use**	**Preparation**
*Brugmansia aurea* Lagerh. SOLANACEAE	*Wandug* or yellow *floripondio*	They are used during *limpia* to treat *shuka*, *mal aire* or *susto*.	A bouquet is made with *Brugmansia* branches, *Santa María de huerta*, *shuyu rosas de cerro*, *laurel grande*, *ruda, marco cari* and *cararango.*
*Brugmansia sanguinea* (Ruiz & Pav.) D. Don	*Wandug* or red *floripondio*	They are used on humans and animals. These species are applied externally.	
SOLANACEAE			
*Brugmansia candida* Pers.	*Wandug* or white *floripondio*	To treat rheumatic pain.	*Brugmansia* leaves are cooked with other plants that are used for the same purposes.
SOLANACEAE			
*Brugmansia suaveolens* (Willd.) Bercht. & J. Presl	*Wandug* or pink *floripondio*	They are taken in baths.	
SOLANACEAE			

When the San Pedro cactus does not have the desired hallucination result, the *Brugmansia* flower is added to induce intoxication; this use has also been reported in Peruvian folk healing rituals [36,39,40]. However, the plant’s use for this purpose among the Saraguro *yachakkuna* is not generalized. According to a Kichwa myth, a beautiful young woman (goddess) was insulted by a vain man; as a vengeance, she placed a powder made of red *wandug* leaves and flowers into a glass of *chicha* and gave it to him to drink. The man lost his voice forever, as this plant is considered very dangerous and may have negative effects if used inappropriately. Muñoz Bernard [22] notes that the *Zhal* indigenous group in the province of Cañar use *chicha* (maize liquor) and *floripondios* liquor to make a person become dizzy and dream like a dead person. This finding may be proof of the plant’s use as a psychoactive specie by other groups in the Andean region of Ecuador.

In a study regarding the use of psychoactive species in Ecuador, Peru, and Bolivia, Kvist [41] reports that the *Brugmansia* spp. are used in place of the San Pedro cactus in Andean regions where the San Pedro cactus is difficult to acquire.

Because of its importance as a medicinal specie in healing rituals, *wandug* is cultivated by *yachakkuna* in their gardens; this is a common practice in various Andean communities [33,41,42].

### Tobacco use

Tobacco (*Nicotiana* spp.) is a sacred specie with an important role in Saraguro rituals. During ceremonies, tobacco can be administered as liquor or it can be inhaled. It is occasionally smoked during the rituals; the tobacco powder obtained from the dry leaf is aspired or blown with inhalers to induce stimulant and sedative effects.

The narcotic and hallucinogenic effect of tobacco has been mentioned in previous studies regarding the traditional medicine of other indigenous groups [43-46]. During these rituals, various species of tobacco are used, including *Nicotiana rustica,* which is called *Sacha* tobacco in the Kichwa language, and *Nicotiana tabacum*. According to *yachakkuna*, *Nicotiana rustica* has a greater psychoactive effect than *Nicotiana tabacum*; this belief is in accordance with the beliefs of traditional shamans from Mexico and South America [45].

**Figure 4:**
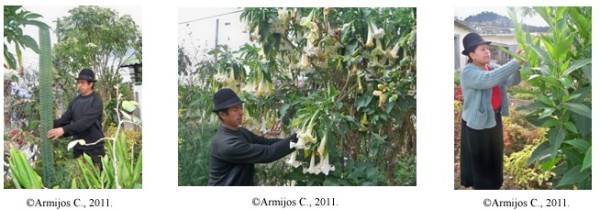
**San Pedro cactus, white *wandug, *and tobacco.** Medicinal species demonstration garden, Saraguro Hospital.

The sacred species most commonly used by Saraguro healers to cure supernatural diseases and in their healing rituals are San Pedro cactus, *wandug*, and tobacco (Figure 4). Psychoactive preparations can be obtained from these plants when used alone or mixed with other vegetable additives. These products can be taken orally or inhaled.

### Nasal administration

According to the aforementioned world view of disease, in the Saraguro community, the nasal absorption of macerated plants has a dual meaning: when absorbed through the right nostril, the plant serves to receive positive energy and to improve the person’s mood; when absorbed through the left nostril, the plant serves to drive out negative energy from the person. Administration by inhalation is faster and more efficient than oral administration because of better absorption in the nasal capillaries; in this manner, the degradation of the substance in the mouth and intestine is avoided [47]. According to previous studies, this practice has been used in the Central Andes for more than 4,000 years [48,49].

### Final considerations

To preserve the traditional Saraguro knowledge about empirical medicine, the Loja Department of Provincial Health and the Healers Council of Saraguro (Consejo de Sanadores de Saraguro) have created a school of traditional indigenous medicine where wise *hampiyachakkuna* share their empirical knowledge and select apt young Saraguro apprentices to continue their training process. In the Saraguro community, knowledge about the uses of sacred and psychoactive medicinal plants is passed orally from generation to generation, mainly from parents to children or a familiar member who is an expert in traditional medicine.

Publications regarding the use of plants in the southern region of Ecuador in indexed journals include Finnerman [9], Béjar, Bussmann [50], Bussmann and Sharon [17], and Cavender and Alban [18]. None of these publications cite the use of psychoactive species for visionary, magical, or religious purposes by the Saraguro *yachakkuna*.

## Conclusions

The current traditional health system in Saraguro community is the cultural expression of the Saraguros’ presence as an Andean group in southern Ecuador: it represents their character as indigenous group, their ability to survive as a community despite strong external pressure, and the desire to maintain their ancient healing heritage.

The modern-day *hampiyachakkuna* represent a synthesis of the ancient knowledge inherited from their ancestors and the knowledge gained during the acculturation process they have undergone. The latter factor represents a distortion of the richness of the original healing system. The disease treatments used in Saraguro communities located far away from the city respect and follow the ancient traditional methods with more rigor than is observed in communities located in urban areas.

It has been confirmed that *yachakkuna* from Saraguro community use psychoactive species, such as San Pedro cactus *Echinopsis pachanoi*, *wandug* (*Brugmansia* spp.), tobacco (*Nicotiana tabacum* L.), and other vegetable additives, during their religious-magical rituals and healing ceremonies to treat physical, mental, emotional, and supernatural diseases. The present study represents a first step in the recovery process of traditional Saraguro medicinal knowledge conducted in collaboration with the Saraguro community *yachakkuna*. It represents a starting point for future studies and state programs that will promote the Saraguro culture and the sustainable development of Saraguro medicinal and biological resources.

## Consent

Permissions were provided by all participants in this study, including the chairman, Council of Healers Orlardo Leonidas Gualán (Figure 2), José Maria Gonzales shown in the photos (Figure 3(A)), Daniel Guamán and Isabel Gualán (Figure 4). They have declared that they have no objection to the publication of their pictures in the journal. The consent was obtained from the participants prior to this study being carried out. The photographers (Daniel Guamán and Chabaco Armijos) transferred the copyrights to the authors.

## Competing interests

The authors declare that they have no competing interests.

## Authors’ contributions

CA conducted the fieldwork. CA, IC, and SG designed the study, performed the data analysis, and drafted the manuscript. All the authors read and approved the final manuscript.
